# Unmanned Aircraft Systems complement biologging in spatial ecology studies

**DOI:** 10.1002/ece3.1744

**Published:** 2015-10-08

**Authors:** Margarita Mulero‐Pázmány, Jose Ángel Barasona, Pelayo Acevedo, Joaquín Vicente, Juan José Negro

**Affiliations:** ^1^ Department of Evolutionary EcologyDoñana Biological StationCSICAvda. Américo Vespucio s/n41092SevilleSpain; ^2^ Sabio IRECNational Wildlife Research Institute (CSIC‐UCLM‐JCCM)IREC. Instituto de Investigación en Recursos CinegéticosRonda de Toledo 1213071Ciudad RealSpain

**Keywords:** Abundance modeling, animal monitoring, biologging, cattle, drones, GPS‐GSM collars, Remote Piloted Aircraft Systems, spatial distribution, Unmanned Aircraft Systems

## Abstract

The knowledge about the spatial ecology and distribution of organisms is important for both basic and applied science. Biologging is one of the most popular methods for obtaining information about spatial distribution of animals, but requires capturing the animals and is often limited by costs and data retrieval. Unmanned Aircraft Systems (UAS) have proven their efficacy for wildlife surveillance and habitat monitoring, but their potential contribution to the prediction of animal distribution patterns and abundance has not been thoroughly evaluated. In this study, we assess the usefulness of UAS overflights to (1) get data to model the distribution of free‐ranging cattle for a comparison with results obtained from biologged (GPS‐GSM collared) cattle and (2) predict species densities for a comparison with actual density in a protected area. UAS and biologging derived data models provided similar distribution patterns. Predictions from the UAS model overestimated cattle densities, which may be associated with higher aggregated distributions of this species. Overall, while the particular researcher interests and species characteristics will influence the method of choice for each study, we demonstrate here that UAS constitute a noninvasive methodology able to provide accurate spatial data useful for ecological research, wildlife management and rangeland planning.

## Introduction

Assessing the distribution of species among available environments and the reasons behind those patterns are recurrent ecological questions that may also affect human activities and conservation efforts (Morrison et al. 2006). Resource utilization, wildlife management, conservation planning, ecological restoration, and prediction of possible future impacts of land use or climate changes are all applied areas that benefit from spatial distribution data of individuals, populations, species, and communities (Collinge 2010; Qamar et al. 2011).

Data for species distribution at large spatial scales are highly demanded in order to establish bases on which management schemes can be sustained, and there is a plethora of methods described for this purpose in the scientific literature (e.g., Seber 1986). For a given species, the effort required to apply each method is highly variable and conditions their applicability to be used mainly at large spatial scales (Acevedo et al. 2008). Obviously, the efforts required to collect data at large spatio‐temporal scales exclusively from fieldwork are unworkable for most of the studies. Thus, surveying a number of representative populations, on which the relationships between species presence/abundance and the environmental conditions can be determined, is a way to forecast the abundance and/or environmental favorability for the species in unsampled territories (e.g., Etherington et al. 2009; Acevedo et al. 2014). In this regard, to record precise information of species distribution is one of the challenges for wildlife management.

Numerous methodologies are available to collect spatial data for animals in the field. Direct methods include observation, capture, phototrapping, biotelemetry and cameras, whereas indirect methods are dependent on some evidence of animal activity (e.g., bed sites, feces, nests, or tracks) (Mcdonald et al. 2012). Biologging consists in the remote data collection from free‐ranging animals using attached electronic devices (Cooke et al. 2004). This is an increasingly popular option among ecologists because it provides valuable information on the animals' movements and habitat use. This method has experienced a remarkable development thanks to the continuous technological advances, especially those regarding tags miniaturization in recent years. Nevertheless, biologging techniques present some constraints, including logistical challenges, possible undesirable effects on the animals during the capture, handling and along the period on which the individuals are tagged (see Murray and Fuller 2000 for a review), and the limitation in the number of animals that can be studied, restricted by the number of tags deployed, which are often expensive (Rutz and Hays 2009).

Unmanned Aircraft Systems (UAS hereinafter) have proven useful to address various ecological challenges involving animal surveys (Jones 2003; Watts et al. 2010; Sardà‐Palomera et al. 2012; Vermeulen et al. 2013) and habitat characterization (Getzin et al. 2012; Koh and Wich 2012). There is a considerable potential value of UAS for spatial ecology (Anderson and Gaston 2013), but to date, there are just a few studies that have explored their possibilities (i.e. Rodríguez et al. 2012; Barasona et al. 2014b). In this context, the aims of this work were to test the suitability of aerial images obtained from UAS flights for i) modeling spatial distribution patterns of animals as compared against a widely used method (biologging using GPS‐GSM collars) and ii) predicting species abundance by comparing estimates from the images with actual abundance in the study area. We use as model species free cattle *Bos taurus* inhabiting Doñana Nature Reserve (Southwest of Spain) under a traditional husbandry system. Cattle are large mammals that offer logistical advantages for biologging deployment and are easily detectable in UAS images. In addition, the knowledge of the spatial distribution of these large herbivores is critical for ecosystem management (Lazo 1995; Bailey et al. 1996). Researchers and park managers are specially interested in cattle spatial distribution because their foraging impact and their interactions with wild ungulates in the protected area constitute a controversial conservationist and sanitary issue (Lazo 1995; Espacio Natural Doñana 2000; Gortázar et al. 2008).

## Materials and Methods

### Study site and species

**Figure 1:**
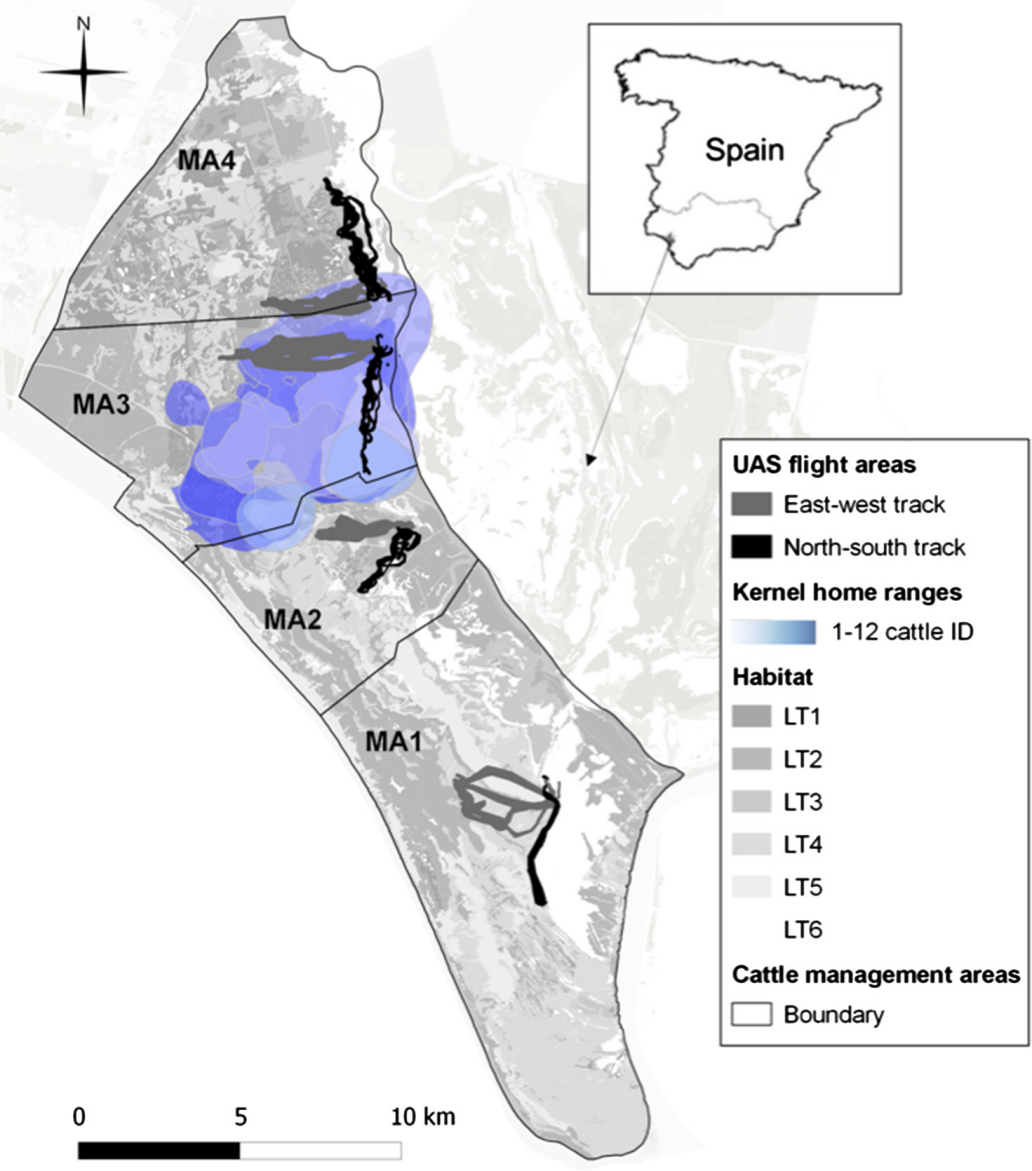
Map of Doñana Nature Reserve study area. Habitat is mainly divided in dense scrub (land cover type, LT1), low‐clear shrub land (LT2), herbaceous grassland (LT3), woodland (LT4), bare land (LT5), watercourse vegetation, and water body (LT6). Unmanned Aircraft System tracks location at the four cattle management areas, and fixed kernel (95% utilization distribution) home ranges of GPS collar locations in the Biological Reserve (MA3) are represented.

Doñana Nature Reserve (DNR hereinafter; 37°0′N, 6°30′W) is located in the right bank of the Guadalquivir river estuary in the Atlantic coast of Southwestern Spain. DNR covers 1008 km^2^ and hosts a variety of ecosystems including marshlands, lagoons, scrub woodland, forests and sand dunes, which led to its declaration as a World Heritage Site and Biosphere Reserve (UNESCO 2014). The area has a Mediterranean climate classified as dry subhumid with marked seasons. We performed the field work during the dry season, when the study area includes the following main habitats (Barasona et al. 2014b): (LT1) dense scrub dominated by *Erica scoparia* and *Pistacia lentiscus*, (LT2) low‐clear shrubland, mainly of *Halimium halimifolium*,* Ulex minor* and *Ulex australis* (LT3) herbaceous grassland, (LT4) *Eucaliptus* sp. and *Pinus* sp. woodlands, (LT5) bare lands, sandy dunes and beaches, and (LT6) water bodies and vegetation associated with watercourses covered mainly by *Juncus* sp. patches (Fig. 1). A north–south‐oriented humid ecotone can be identified between the scrublands and the edge of the dry marshlands (Barasona et al. 2014a), dominated by *Scirpus maritimus* and *Galio palustris* with *Juncus maritimus* associations. The study area in DNR is divided into four management areas (MAs hereinafter) from south to north named, respectively: Marismillas (MA1), Puntal (MA2), Biological Reserve (MA3), and Sotos (MA4).

Our model species is free‐ranging cattle *Bos taurus* that occupy different MAs along the protected area. The cattle management has traditionally been minimal, with the animals being captured just once per year for sanitary handling. Doñana cattle is mainly an autochthonous breed, named “Mostrenca,” although some cross‐breeds exist in some herds. This cattle population is especially interesting from an ecological perspective because free‐ranging cattle with little human interference is not common in large protected Mediterranean ecosystems (Lazo 1995). Since 2000, cattle are managed according to the Cattle Use Plan (Espacio Natural Doñana 2000) which determines the maximum number of individuals allowed on each MA. The cattle numbers provided by the DNR authorities for this study dates were MA 1 = 318, MA 2 = 152, MA 3 = 168, and MA 4 = 350 and are based on the annual sanitary campaign (July 2011) where all the animals are captured and identified by ear tags.

### Unmanned Aircraft Systems (UAS) methodology

We completed a total of 192 km of UAS diurnal aerial tracks of two types (east–west‐ and north–south‐oriented transects) on each cattle management area with six replicates (Fig. 1). UAS surveys took place during August and September 2011, the end of the dry season and a time when food resources become more limiting for herbivores in DNR in terms of water and forage availability (Bugalho and Milne 2003) between 15.00 h and 20.00 h local time. The tracks were performed at an average speed of 40 km/h at 100 m altitude above ground level. The covered strips were approximately 4 km long and 100 m wide (Fig. 1).

**Figure 2:**
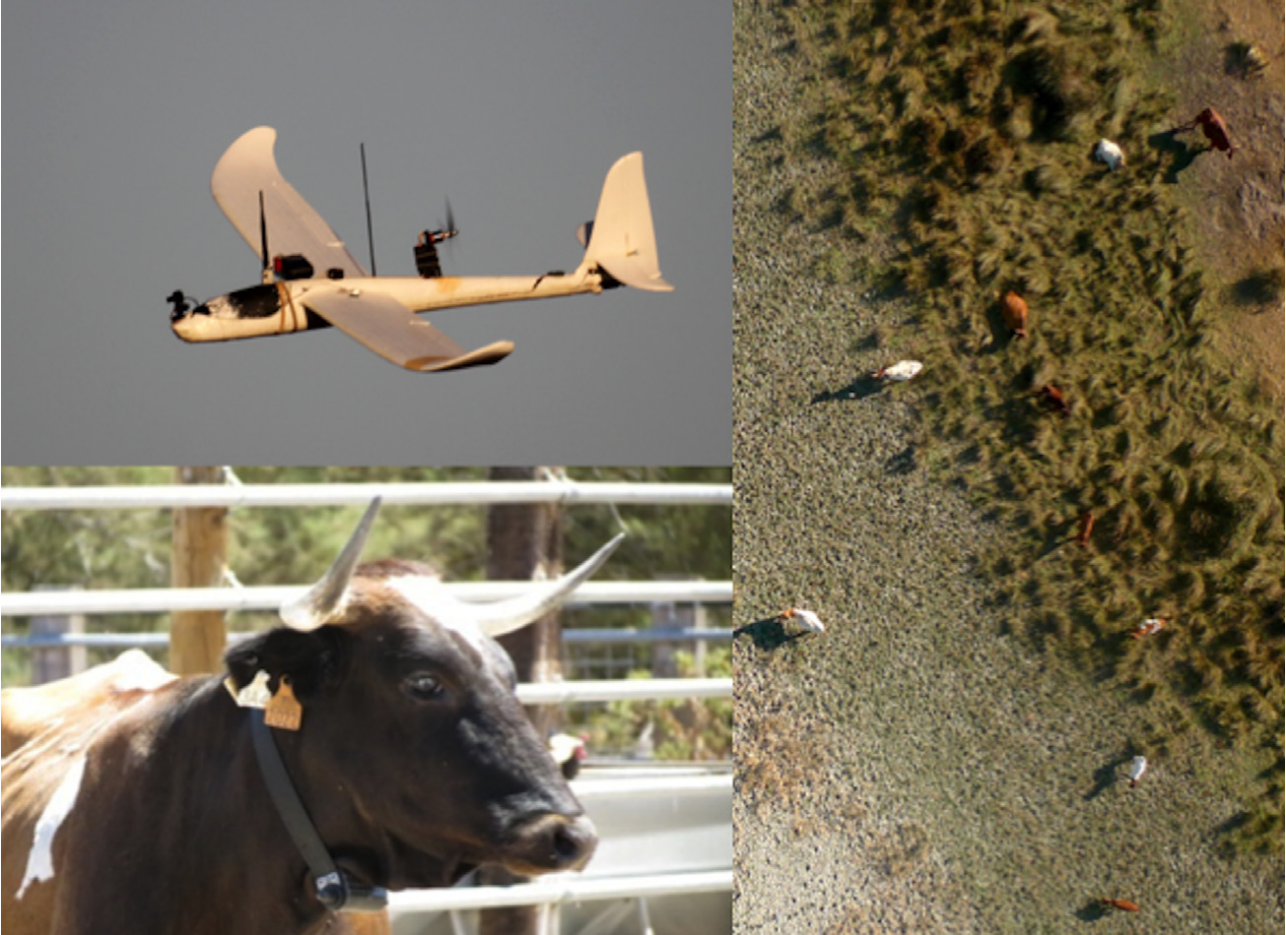
Left: Unmanned Aircraft System (UAS). Mostrenca cattle equipped with GPS‐GSM collar. Right: image obtained with UAS of Mostrenca cattle aggregated in the ecotone of the study area.

The flights were carried out with a small UAS (1.96 m wingspan; see Fig. 2) assembled at Doñana Biological Station using a foam fuselage of an Easy Fly plane (St‐models, Jiaxing, China) propelled by an electrical engine. It is equipped with an Ikarus autopilot (Electronica RC, Seville, Spain), which provides waypoint following capability and an Eagletree GPS logger V.4 (Eagletree systems, Bellevue, WA) with a barometric altitude sensor. The digital photo camera Panasonic Lumix LX‐3 11MP (Osaka, Japan) is integrated in the plane wing nadir pointing, and the shutter is activated by a mechanical servo. The images were taken in speed priority mode and in its widest zoom position with continuous shooting. Total price of the system was around 5700 € as of June 2011.

We georeferenced the images using the information provided by the UAS and Eagletree data with a customized extension of ENVI software to produce GeoTIFF files. Accuracy of our UAS locations is estimated in the range of 10–50 m before postprocessing (Mulero‐Pázmány et al. 2014a,b; M. Mulero‐Pázmány, unpublished data) and was improved up to 1–3 m after GIS corrections (superimposing the images on orthophotographs and manually correcting them using reference points). We traced the animals in the images and processed them over a 1 ha‐approximated patch size (grid) as proposed in detailed studies on ungulate behavior (Gibson and Guinness 1980).

### Biologging methodology

Twelve adult female Mostrenca cattle selected randomly from different familiar groups were equipped with GPS‐GSM collars in July 2011 in the Biological Reserve (MA3) (Fig. 2) during routine veterinary inspections with the animals restrained in a cattle chute. The collars included a satellite position capture system (GPS) and a Global System for Mobile communications (GSM) (Microsensory System, Spain) (Cano et al. 2007). The price per collar is 2750€ plus sms service, covered by the manufacturers in our case. The collars were programmed to take a GPS location every hour, sending encoded packets with 20 positions to the central station when mobile phone coverage allowed. Data collected included the following: date, time, geographic coordinates, and location acquisition time (LAT hereinafter, precision measure to obtain a fix; range from 0 to 160 sec). We screened our data using LAT ≥ 154 sec to detect anomalous fixes (manufacturer's technical data; Microsensory System, Spain). We obtained a fix rate of 93.95%, which is acceptable considering that fix‐rate success of <90% can cause habitat‐induced bias in resource selection studies (Frair et al. 2004). Positional error associated with GPS locations was 26.64 m on average, SD = 23.5 m, according to stationary tests carried out in the center of our study area.

### Data analysis

#### Landscape covariates

Environmental variables were estimated from thematic cartography 1:10,000 scale (Consejería de Medio Ambiente y Ordenación del Territorio. 2013) using Quantum GIS version 1.8.0 Lisboa (QGIS Development Team 2012) and were determined following the information of the landscape factors potentially driving ungulates spatial distribution in the study area and related to habitat quality (Braza and Alvarez 1987; Lazo 1995; Barasona et al. 2014b). For each 1 ha grid of the study area (total = 29,532 grids, including the 10.1% corresponding to UAS track grids; *n* = 2983; 3728.75 ha) and for each 26 m radius buffer (according to GPS positional error (Recio et al. 2011)) around each GPS used and available cattle locations (Jerde and Visscher 2005), we calculated the following: distance to nearest artificial water hole (DW), distance to nearest marsh‐shrub ecotone (DE), exact grid area (GA) to control the variation in UAS image areas in the case of UAS track grids, and proportion of the different land cover types (LT1‐LT6). Distances, areas, and land cover type proportions were treated as continuous variables (Table S1), and cattle management area (MA), as a categorical variable. Distance variables were obtained as the shortest distance from each grid and buffer centroid to the nearest environmental feature.

To correct visibility reduction produced by vegetation cover for cattle detection in UAS images, we calculated detection coefficients for LT1 and LT4 land cover types. We estimated the detection proportion of 100 random circle points (1 m^2^ size) created in QGIS from ten different habitat images (1 ha) of each cattle management area and land cover type (80 images analyzed) considering any point above vegetation cover as “not detected” and any point without vegetation cover as “detected” (Barasona et al. 2014b). Detection coefficients used in statistical analysis were 0.544 for LT1 and 0.360 for LT4, respectively. Colinearity between explanatory variables was tested with Spearman's pairwise correlation coefficients *r* > ¦0.5¦ (Hosmer and Lemeshow 2000).

#### Cattle distribution modeling

We tested the factors affecting the spatial distribution of cattle (1) using UAS images as a first approach and (2) using GPS‐GSM collar locations as a second approach, by means of generalized linear models (GLM).

For the UAS model, we only included the east–west UAS track data, because north–south UAS tracks showed low habitat feature variation (these data were later used for model validation). The response variable was the number of detected animals per UAS grid and was modeled with a negative binomial distribution and logarithmic link function (Cameron and Trivedi 2013). The final UAS model was obtained using a backward stepwise procedure based on the Akaike information criterion (AIC) (Akaike 1974).

For the GPS model, we used resource selection function (RSF) logistic regression (Manly 2002) where used locations (only considering the ones obtained during the same period hours of UAS flights) were coded as 1, and random locations (available, ten per used GPS location), inside the individual fixed kernel (95% utilization distribution) home ranges, as 0. The response variable was the presence/absence of cattle in the grid, and the model included the variables selected for UAS approach except the MA categorical factor (as the collared animals were restricted in MA3). Residuals of both UAS and GPS models were examined and tested for spatial autocorrelation using the Moran's I in order to detect spatial structures (Diniz‐Filho et al. 2003).

#### Validation and comparison between the two methods

UAS model validation was performed by mean of Pearson' correlations with independent (20%) data of the east–west tracks and all information in north–south UAS track dataset. GPS model validation was performed by assessing the predictive capacity of each model with the area under a relative operating characteristic (ROC) curve (AUC), to rate the probability that the models correctly discriminated between used and random locations. The AUC ranges from 0.5 for models with no discrimination ability to 1 for models with perfect discrimination (Pearce and Ferrier 2000). Spatial predictions of both final models were transferred to MA3 area where visual and quantitative comparisons were conducted to verify correspondence between predictions of UAS and GPS approaches by Spearman's pairwise correlation. All statistics were performed in R version 3.0.1 (R Development Core Team 2013).

We also compared the densities (number of animals/surface) predicted by the UAS model with the actual density in the different MAs (data provided by Doñana Biological Reserve and Doñana National Park authorities for the studied time period) and evaluated cattle aggregation in the grids by variance to mean ratio (Elliot 1977).

## Results

A total of 358 individual cattle were identified and located on the UAS track images along DNR (Fig. 2). We did not observe any disturbance reactions to the UAS during the overflights from the cattle nor from other ungulates present in the area. Overall, the GPS collars fixed 1752 locations of the 12 marked animals during the same period of UAS flights. Table S1 illustrates the descriptive statistics for the analyzed continuous landscape covariates in the UAS track grids, GPS (used and available) location buffers, and total MA3 and DNR grids.

Results of the variables included in the spatial distribution models selected by the stepwise procedure (ΔAIC), estimated coefficients, standard errors, and significance are summarized in Table 1 for each approach. The best fitting UAS model (AIC = 397, ΔAIC from saturated model = −32) found that the environmental covariates influencing cattle distribution are mainly related to land cover types, with a positive effect of grasslands on the ungulates distribution and a negative effect of the distance to the ecotone and shrubs. The best fitting UAS model also revealed a significant effect of the management area on cattle abundance. GPS method identified all the included variables as significant and showed a similar effect of them over cattle presence.

**Table 1:** Results of generalized lineal models to determine the most relevant factors explaining cattle distribution patterns in Doñana Nature Reserve: Best fitting model for Unmanned Aircraft System (UAS) approach (response variable is “number of detected animals in 1 ha grid”) and a model for biologging (GPS collars) with UAS‐selected covariates (response variable is “presence/absence in a 1 ha grid”). Estimated coefficients and standard errors (SE) are shown

		Estimated coefficients (SE)	
		UAS method	GPS method
Intercept		−2.6910 (0.7280)***	−0.0820 (0.0610)
Variables			
DE	Distance to nearest marsh‐shrub ecotone (km)	−0.0006 (0.0004)*	−0.0028 (0.0001)***
LT1	Dense scrub (%)	−13.270 (4.3270)**	−0.0206 (0.0011)***
LT2	Low‐clear shrub (%)	−2.0360 (0.86189*	−0.0316 (0.0013)***
LT3	Herbaceous grassland (%)	2.3320 (0.6438)**	0.0044 (0.0007)***
MA1	Management area (1)	Ref. category	
MA2	Management area (2)	2.8060 (0.7901)***	
MA3	Management area (3)	1.8070 (0.8591)*	
MA4	Management area (4)	2.2570 (0.9636)*	

*P* values: *P < 0.05, **P < 0.01, ***P < 0.001.

**Figure 3:**
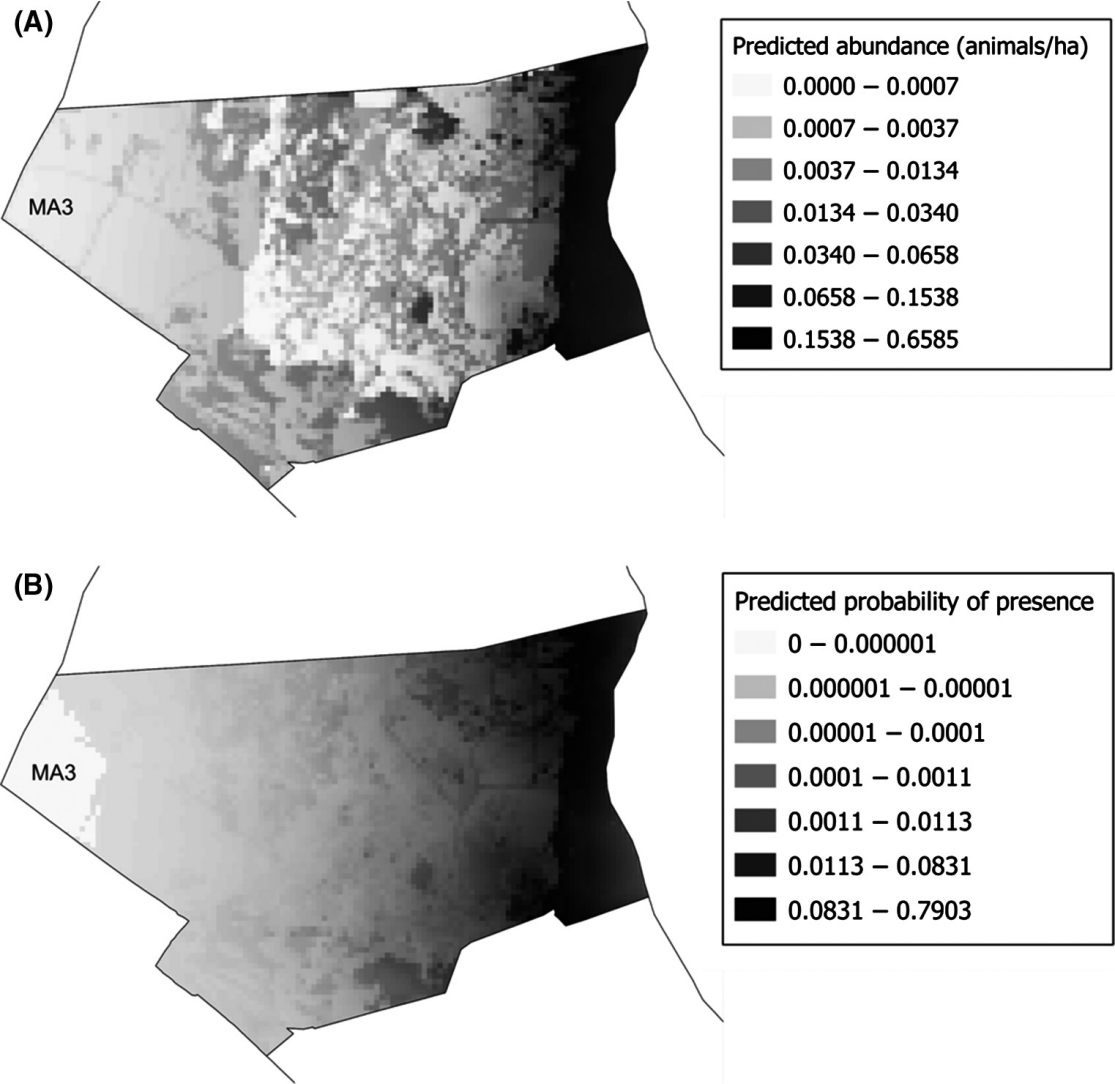
Map of Doñana Biological Reserve study area (MA3) with the transference at 1 ha spatial resolution of the cattle predicted spatial distribution values obtained by modeling landscape variables with: (A) Unmanned Aircraft System (UAS) dataset (predicted abundance of animals); and (B) Biologging (GPS‐GSM collars) dataset (predicted probability of presence).

Validation of the model predictive performance on independent UAS track datasets showed that the selected best spatial distribution model performed with significant Pearson's rank correlations (east‐west data: *r* = 0.30, *P* < 0.001, *n* = 258; and north–south data: *r* = 0.32, *P* < 0.001, *n* = 852). The assessment performed for the GPS location model showed a high predictive capacity (AUC = 0.945). The residuals of both models were not spatially structured according to Moran's I index. These validation results permitted the transference of the models to the MA3 using total 1 ha grids (Fig. 3).

The map representing predicted spatial distribution of cattle shows common distribution patterns throughout MA3 between UAS and GPS approaches. High relations were found between the predicted values of UAS and GPS methods in the MA3 by Spearman's rank correlation: *r* = 0.716, *P* < 0.001, *n* = 6501.

The mean of predicted densities calculated by the UAS approach for each MA was higher than the densities provided by DNR authorities, showing differences between the four MA of DNR, with more overestimated values in the MA with higher aggregation coefficients (Table 2).

**Table 2:** Comparison of actual cattle density (individuals/ha) in four different management areas in Doñana Nature Reserve with predicted density calculated with Unmanned Aircraft Systems dataset. Variance to mean ratio as an aggregation indicator

Management area	Actual density	UAS predicted density	Predicted to actual density ratio	Variance to mean ratio
1	0.031	0.035 ± 0.030	1.13	1.77
2	0.040	0.118 ± 0.124	2.95	19.82
3	0.026	0.033 ± 0.084	1.27	2.79
4	0.057	0.139 ± 0.196	2.44	15.84

## Discussion

In an effort to assess the ability of UAS to contribute to animal spatial ecology studies, we compared the predicted spatial patterns of free‐ranging cattle in Doñana Biological Reserve obtained using animal locations from UAS overflights images against locations from biologged cattle (GPS‐GSM collars). Both models, using the same environmental covariates, performed well and provided similar spatial distributions of cattle at a very fine scale (1 ha grids).

### Model results

The environmental variables selected by the UAS model to explain the abundance of cattle are those expected to be more important from an ecological perspective. The positive influence of herbaceous grasslands on ungulates distribution reflected by our models has been previously identified (Bailey et al. 1996) indicating the need to forage on green pastures during the dry season. Previous work conducted specifically in our study area also indicated that seasonality in the phenology of the herbaceous layer has major repercussions in the ranging behavior of Doñana cattle (Lazo 1995) that concentrate in the areas identified by our models when the resources are scarce. The ecotone between the shrublands and the marshlands is the higher quality habitat of DNR, offering perennial grasses with high levels of palatability (Lazo 1995). This area keeps a high soil humidity and offers not only grasslands but also tree shade and refuge which are valuable for ungulates in the dry season (Braza and Alvarez 1987). Models also showed a negative effect of dense and low‐clear shrub on cattle presence that tend to avoid those land types in favor of the open grassland areas (Casasús et al. 2012). However, as this work is limited to data obtained at a specific season and time of the day, because our main goal is to compare two methods in the same conditions, general habitat use by cattle should be addressed in a more complete study performed all year/day round.

Although the UAS method worked successfully for predicting cattle spatial patterns, it overestimated cattle density in all the management areas (Table 2). This discrepancy may be explained because the flight locations were biased toward the areas where cattle is more concentrated, a problem which could be solved by performing stratified surveys in the different habitats. Also, the overestimation is not homogeneous along DNR, but higher in those areas with a more aggregated distribution. This fact has been proven relevant for animal surveys in general and manned aerial censuses – more related with UAS – in particular (Tellería 1986; Fleming and Tracey 2008). There are various protocols to assess this effect (Redfern et al. 2002; Tracey et al. 2008) and techniques to correct it (Bayliss and Yeomans 1989; Fleming and Tracey 2008) that should be considered if the researcher main objective was estimating abundance, for instance increasing sampling effort as cattle spatial aggregation does.

### Methods comparison

Although biologging and UAS approaches proved to be useful in our study, there are several factors that condition their general applicability in spatial ecology. The most desirable aspects for carrying out spatial ecology studies are to optimize sampling size and data accuracy while maximizing diversity and frequency. However, it is also required to minimize impact, cost, logistic, and data‐processing effort. On these bases, we provide below an analysis of the pros and cons of each method.

#### Sampling size

Sampling size for biologging is limited by financial constrains and/or trapping success (Cooke et al. 2004; Rutz and Hays 2009; Hebblewhite and Haydon 2010). This may lead to incurring in data biases caused by the selection of animals to be fitted with tags, including that produced by the non‐random selection in relation to age, sex, and geographic location, which increases if the trapping method is not selective. Deployed tags can fail because they may stop sending data or becoming lost, further reducing sample size, a fact that may lead to biased inferences by focusing on the space use of a few individuals while ignoring the position of nontagged animals (con‐ or heterospecifics).

Sampling size for UAS monitoring depends in the first place on the area the system is able to cover during the flights (which in turn depends on UAS range and autonomy) and secondly on UAS detection capacity. Both factors are related and UAS flight altitude must be a compromise between obtaining adequate resolution to distinguish the species under investigation and the size of the area to cover.

Fleming and Tracey (2008) analyzed the efficacy of manned aerial surveys, which is also applicable to UAS, identifying the size, shape, color, shadow (which can be related to time of the day), and contrast against background of the animals, as well as their response to the aircraft, as relevant factors for detection. Our experiments were conducted with cattle that present large size and color patterns, offering high contrast with the surrounding vegetation, and performed in the late afternoon; thus, those factors seemed irrelevant. We easily spotted cattle adults and calves, along with other ungulates such as wild boars, red and fallow deers, with the embarked 11 MP commercial camera flying at 100 m altitude above ground level. Smaller animal such as birds have also been detected in daylight conditions from UAS (e.g., Sardà‐Palomera et al. 2012) although flying at lower altitudes.

Species behavior and habitat characteristics also affect detectability by means of UAS. Bayliss and Yeomans (1989) noted that the main source of (manned) aerial survey bias of feral livestock is obstructive vegetation cover. We addressed this problem in our study using detection coefficients adequate for the present land covers. This coefficient, estimated from random location of points, assumes that animals are also randomly distributed with respect to tree cover, but if the animals were actively seeking tree cover, then the densities obtained by UAS could be underestimated, or just the opposite if individuals selected otherwise. Besides, selection for cover may vary among species, individuals, season, and time of day (in our case, all the flights were performed in the late afternoon and during summer). Equipping UAS with thermal cameras allows distinguishing animals in dense vegetation areas or at night. Nevertheless detectability and animal identification with thermal cameras can be difficult for daylight conditions and in dense vegetation habitats (Mulero‐Pázmány et al. 2014b).

It is important to consider that any of the above mentioned physical or behavioral characteristics that influence UAS detectability may affect differentially a subgroup of the target species (such as a sex or age classes), which could potentially bias spatial ecology studies conducted with UAS. Admittedly, physical characteristics, behavioral responses, and habitat features are less critical when data are obtained through biologging. On the other hand, assuming a suitable detection rate for UAS, one of the main advantages of this method versus biologging is that it provides the researcher with an image of the animals that are present in the area, permitting to include group influence or interspecific aggregation as variables of the ecological studies.

#### Data accuracy, diversity, and frequency

Spatial accuracy of the animal locations obtained by UAS after processing is estimated between 1 and 3 m. This constitutes a major advantage for UAS in spatial distribution studies against biologging that provides less accuracy (e.g., 26 m for the GPS collars we used).

The use of specific sensors in biologging tags is developing fast, allowing to measure individual parameters (e.g., physiological, behavioral, movement speed and range), which is information that could not be obtained with the UAS approach. On the other hand, UAS have the capacity to provide real‐time information on habitat characteristics, which is especially interesting in highly dynamic landscapes (Rodríguez et al. 2012), where short‐term changes affecting animals' movements (i.e., produced by fires, human interventions and flooding) may not be reflected on satellite or GIS resources available with proper spatial–temporal resolution. This temporal accuracy is a major advantage, as obtaining animal information and environmental variables at the same level of detail and reliability would significantly improve ecology studies (Gaillard et al. 2010; Hebblewhite and Haydon 2010).

While trapping animals may be complex, once the animals are biologged, they can produce enormous volumes of data for a long period of time. In contrast, to obtain long‐term data with UAS would require numerous flight field campaigns, and with this method, it is difficult to identify specific individuals in the images and recognize them on subsequent flights.

#### Impact

Biologging requires capture and handling of the animals that besides involving bioethical approval might affect their behavior and survival (Silvy et al. 2012), thus complicating the use of this technique (Cooke et al. 2004). A point in favor of the use of UAS is that due to the small size and the reduced noise that these systems produce, animal response is very low (at least not visually noticeable in our case) so that the method does not significantly disturb the study subjects. Electric UAS are also zero‐emission vehicles, and this is an aspect particularly important when surveying nature reserves. Additionally, because UAS are classified as a noninvasive technique, no approval by animal committees is deemed necessary, but legal constraints may affect their use in countries with strict aerial regulations that can prevent the use of this approach.

#### Cost, logistics, and data‐processing effort

We invested 33000 € in the 12 cattle collars used for this work. In contrast, the complete UAS we used had a cost of 5700 €. As a reference, using data from the same time period in our study for both methods, we obtained single locations of 358 cattle with UAS flights (2615 ungulates located in total: horses, red and fallow deer, and wild boar) versus the 1752 locations of 12 cattle individuals that were marked with radiocollars. Data retrieval is simple for GPS‐GSM biologging systems, as the researcher receives animal locations at this office, but the UAS method requires images postprocessing (georeferencing and detecting the animals in the images) which in our case took about 40 h of work.

In summary, our results demonstrate that UAS constitute an effective tool for spatial ecology by providing the data required to develop distribution models for at least large animals, which may be comparable to those obtained using other widely accepted techniques such as biologging. Different methodologies have their own strengths and weaknesses, so the decision on which one to use would depend on the researcher objectives. We foresee that a combination of both approaches may prove an interesting synergy. UAS can be a complementary method to broaden objectives in animal spatial studies or to include more spatially and/or socially representative samples. For instance, UAS could be used to obtain a first general picture of a species spatial distribution and abundance patterns that could later be used to select the areas and/or individuals more adequate to capture for biologging. Additionally, information of intra‐ and interspecies interactions for larger groups obtained by UAS could be combined with fine detailed habitat selection data obtained from fewer biotagged individuals (or obtained by other methods).

### Management implications

The cattle predictive models obtained in this study contribute to a better understanding of the free‐grazing herbivore distribution patterns within a protected area, which is critical for ecosystem management (Bailey et al. 1996) because these species have spatially variable impacts on resources (Gordon 1995). Individual or groups contact patterns at intra‐ or interspecific levels, and the study of interactions with habitat features (e.g. environmental aggregation points such as water points) is also crucial for evaluating the epidemiology of diseases in the wild, for which UAS provided excellent information (Barasona et al. 2014b). The methodology developed for this study is not only useful for ecology, wildlife, and epidemiology research, but also for rangeland managers who need livestock accurate information for designing effective strategies to optimize their resources (Coulombe et al. 2006).

## Conflict of Interest

None declared.

## Supporting information


**Table S1.** Environmental covariates, descriptions, mean values (X) and standard deviations (SD) of UAS track grids and GPS locations buffers versus MA3 and total study area grids used in the analysis of cattle spatial abundance patterns in Doñana Nature Reserve (DNR).
