# Supplementary material for: Unmanned Aircraft Systems complement biologging in spatial ecology studies
Source: Ecol Evol. 2015 Oct 8;5(21):4808–18. doi: 10.1002/ece3.1744 (PMC4662332; doi:10.1002/ece3.1744)
Supplement: Supplementary file 1 — Table S1. Environmental covariates, descriptions, mean values (X) and standard deviations (SD) of UAS track grids and GPS locations buffers versus MA3 and total study area grids used in the analysis of cattle spatial abundance patterns in Doñana Nature Reserve (DNR). [file ECE3-5-4808-s001.docx]

**Supplementary material**

Table S1. Environmental covariates, descriptions, mean values (X) and standard deviations (SD) of UAS track grids and GPS locations buffers versus MA3 and total study area grids used in the analysis of cattle spatial abundance patterns in Doñana Nature Reserve (DNR).

| Code | Variable | UAS grid (X±SD) | GPS location buffer used and available (X±SD) | Total MA3 (X±SD) | Total study area (X±SD) |
| --- | --- | --- | --- | --- | --- |
| DW | Distance to nearest water point (km) | 0.33±0.19 | 0.55±0.38 | 0.94±0.79 | 0.97±0.73 |
| DE | Distance to nearest marsh-shrub ecotone (km) | 1.22±0.90 | 1.50±1.49 | 4.06±2.76 | 2.47±2.17 |
| GA | Exacted UAS grid area (ha) | 1.25±0.85 | 0.21±0 | 1±0 | 1±0 |
| LT1 | Dense scrub (%) | 24.66±35.20 | 20.95±36.1 | 18.50±31.54 | 11.09±26.28 |
| LT2 | Low-clear shrub (%) | 27.54±34.37 | 31.14±42.15 | 48.59±41.54 | 32.04±39.89 |
| LT3 | Herbaceous grassland (%) | 14.25±26.79 | 28.67±40.94 | 10.92±26.23 | 12.34±26.82 |
| LT4 | Woodland (%) | 18.40±34.24 | 4.16±18.27 | 11.14±26.28 | 19.84±34.77 |
| LT5 | Bare land (%) | 8.82±24.41 | 2.55±12.93 | 4.58±15.71 | 11.30±26.88 |
| LT6 | Watercourse vegetation (%) | 6.33±19.95 | 12.28±30.03 | 4.50±17.61 | 11.28±28.24 |
| MA | Cattle management area (categorical 1-5) | **-** | - | **-** | **-** |
